# Insight Into Dynamics of Gut Microbial Community of Broilers Fed With Fructooligosaccharides Supplemented Low Calcium and Phosphorus Diets

**DOI:** 10.3389/fvets.2019.00095

**Published:** 2019-03-29

**Authors:** Sanjay Kumar, Yue Shang, Woo Kyun Kim

**Affiliations:** ^1^Department of Poultry Science, University of Georgia, Athens, GA, United States; ^2^St. Boniface Hospital Research Centre, Winnipeg, MB, Canada

**Keywords:** calcium, phosphorus, fructooligosaccharide, gut microbiome, chicken

## Abstract

We investigated how the microbiota in the ileum and cecum of broiler chickens fed a diet of low calcium (Ca) and available phosphorus (aP) and prebiotic fructooligosaccharides (FOS) supplements changed over a 3 weeks period. Three dietary treatments were randomly assigned to four replicate cages of five birds each, including: positive control (PC), a wheat-corn-soybean meal-based diet; negative control (NC), as PC with 0.2% reduced Ca and aP; and NC + FOS, as NC supplemented with 0.5% of FOS. Ileal and cecal digesta were sampled from each replicate (*n* = 4) on d21 and processed for 16S rRNA gene amplicon (V4 region) sequencing using Illumina platform. Statistical differences were observed in the microbiome by GI location as determined by 2-way ANOVA and Permutational MANOVA. On average, 24,216 sequence reads per sample were generated resulting in 800 and 1,280 operational taxonomic units in the ileal and cecal digesta, respectively. Difference (*P* < 0.0001) on alpha diversity and abundances of several phyla was observed between ileal and cecal digesta. ß-diversity was different (*P* < 0.05) between each treatment groups in the ileum but not in the cecum. In the cecum, species richness, phylogenetic diversity, and the number of observed species were higher in PC compared to NC + FOS (*P* < 0.05). Several phyla, including Cyanobacteria, Firmicutes, and Proteobacteria, had significantly different abundance in the ileal and cecal digesta (*P* < 0.05). In the ileal digesta, positive correlation were observed between *Salinibacterium* and *Lysobacter* and PC diet. *Blautia, Faecalibacterium* and *Pseudomonas* and the NC diet and *Lactobacillus* and *Escherichia* and the NC + FOS diet. In the cecal digesta, *Butyrivibrio*, and *Allobaculum* were positively correlated to PC. Although, *Clostridium* and *Anaerotruncus* were positively correlated to NC + FOS, they showed negative correlation to PC and NC. The study concludes that dietary Ca and aP level and FOS supplementation alters ileal microbiota of the broiler chickens.

## Introduction

Phosphorus (P) is an essential macro-element in broiler diets and is responsible for multitude of physiological processes including skeletal system development, growth, and productivity of birds. Plant-based diets contain large amounts of unavailable P, which is partially hydrolyzed by the monogastric animals including broiler chickens (1). Therefore, in a standard poultry diet formulation inorganic P supplementation is required to ensure that the available P (aP) meets the requirements for poultry. Changes in the Ca:aP ratio of the broilers diet can affect physio-chemical properties of the digesta in the gastro-intestinal tract of the broilers (2). Increase of the crop (4.89–5.32) and ileum (6.62–7.39) pH by increasing dietary Ca from 10.7 to 25.3 g/Kg was reported by Shafey et al. (3). A negative correlation between broiler performance and the increased pH of the small intestine, by increasing of Ca level in the diet was reported by McDonald and Solvyns (4).

Fructooligosaccharides (FOS), obtained from plants, are non-digestible carbohydrates known to have prebiotic properties (5, 6). Several studies reported that supplementation of FOS has resulted in increased body weight and decreased feed conversion ratio of the birds (7–9). However, performance can be affected by variations in the levels of FOS supplementation. To date, no well-defined recommendation of dietary FOS supplementation is available for poultry. Apart from enhancing the bacterial fermentation in the intestine (10–12), there are also increasing evidence on the potential ability of FOS to increase the bioavailability of minerals. According to Xu et al. (7), 0.4% of FOS in a diet had substantial positive effects on intestinal morphology which might resulted in improved mineral absorption in broilers. Several studies have also proved that supplementation of FOS enhanced the growth of bacterial with probiotic properties (resistance to gastric pH and bile, adherence to mucus/epithelial cells) such as Bifidobacteria and Lactobacilli. These probiotic bacteria produce short-chain fatty acids which result in a lower pH of the gastrointestinal tract [GIT; (13, 14)]. A lower pH has been reported to be favorable for mineral solubility (15, 16). Furthermore, changes in the pH of GIT may result in shifts of the microbiota and their functional attributes. A lower pH in the GIT, due to its bacteriostatic effect, should have negative effect on the population of foodborne pathogens like *Salmonella, Campylobacter*, and *Escherichia coli*, while maintaining the integrity of the intestinal membrane (17). Hence, we were intrigued about microbial community dynamics in gastro intestinal tract of broilers in response to FOS supplemented diet containing reduced Ca and P levels. As very little data are available on the effect of low calcium, phosphorus and FOS supplementation on the microbiome of the host. Therefore, the aim of the present study was to investigate effects of dietary Ca and aP level and FOS supplementation on microbiota in the ileum and cecum segments of the broilers.

## Materials and Methods

### Birds and Housing

A total of 60, 1-day old, male Ross × Ross 308 chicks were obtained from a commercial hatchery (Carltons Hatchery, Grunthal, Manitoba, Canada). The chicks were housed in electrically heated Jamesway battery brooders (James Mfg. Co., Mount Joy, PA) for the first 4 days of pre-experimental period. The temperature during pre-experimental period was 32°C. On d 5, chicks were individually weighed and sorted into five weight classes. Groups of five birds, one from each weight class, were then randomly assigned to 12 battery pens such that the average initial BW was similar across pens. The chickens were raised for 21 d. During the experimental period, birds were housed in three electrically-heated Alternative Design Super Brooders (Alternative Design Manufacturing and Supply, Inc., Siloam Springs, AR) under a controlled environment. The temperature was monitored daily and was gradually reduced until a temperature of 24°C was reached on d 21. Light was provided for 24 h throughout the experimental period.

### Dietary Treatments

Three dietary treatments were randomly assigned to four replicate cages of five birds each. Composition and analyzed nutrient values of the experiment diets are shown in Supplementary Table 1. The dietary treatments include: a positive control (PC), a wheat-corn-soybean meal-based diet contained adequate levels of Ca and available P (aP); a negative control (NC), as PC with 0.2% reduced Ca and aP; and a NC + FOS, as NC supplemented with 0.5% of FOS. The PC diet was fed to all the chickens for the first 4 d adaption period, and the experimental diets were provided from d 5–21. Water and feed were offered *ad libitum*. The basal diet was formulated to meet or exceed the National Research Council nutrient requirements for broiler chickens (18).

### Sample Collection and DNA Extraction

On d 21, a total of 12 birds (one bird from each pen; four birds per treatment) were randomly selected and euthanized by cervical dislocation. Ileal and cecal samples were collected in sterile bags and snap frozen in liquid nitrogen and later stored at −80°C till further processing. The archived ileal and cecal samples were thawed and subjected to DNA extraction using ZR fecal DNA MiniPrep Kit (ZYMO Research, Irvine, CA). Before following the kit protocol, microbial cells were subjected to bead beating using Mini-BeadBeater-16 (Bio Spec Products, Bartlesville, OK) for 2 min. Extracted DNA was the quantified using a NanoDrop 2000 spectrophotometer (NanoDrop, Wilmington, DE, USA). DNA samples were normalized to 20 ng/μL, and quality was checked by PCR amplification of the 16S rRNA gene using universal primers 27F (5′ -GAAGAGTTTGATCATGGCTCAG-3′) and 342R (5′ -CTGCTGCCTCCCGTAG-3′) as described by Khafipour et al. (19). Amplicons were verified by running on 1.5% agarose gel.

### Bioinformatics Analyses

Bioinformatic analyses were performed as described by Derakhshani et al. (20). In brief, the PANDAseq assembler (21) was used to merge overlapping paired-end Illumina fastq files. All the sequences with mismatches or ambiguous calls in the overlapping region were discarded. The output fastq file was then analyzed by downstream computational pipelines of the open source software package Quantitative Insights into Microbial Ecology [QIIME, (22)]. Assembled reads were demultiplexed according to the barcode sequences and exposed to additional quality-filters so that reads with more than 3 consecutive bases with quality scores below 1e^−5^ were truncated, and those with a read length shorter than 75 bases were removed from the downstream analysis. Chimeric reads were filtered using UCHIME (23) and sequences were assigned to Operational Taxonomic Units (OTU) using the QIIME implementation of UCLUST (24) at 97% pairwise identity threshold. Taxonomies were assigned to the representative sequence of each OTU using RDP classifier (25) and aligned with the Greengenes Core reference database (26) using PyNAST algorithms (27). The OTUs that classified to kingdom Archaea were removed from downstream analysis. Venn diagrams [VENNY; (28)] were produced based on classified and unclassified genera obtained from the Greengenes Core reference database, demonstrating the number of shared and unique genera across the PC, NC, and NC + FOS dietary treatments.

Within community diversity (α-diversity) was calculated based on OTU counts using QIIME to evaluate the biodiversity of the bacterial population at the genus level. Alpha rarefaction curve was generated using Chao 1 estimator of species richness (29).

### Statistical Analysis

The α-diversity, major phylum, and genus percentage data from the microbiome sequencing were also analyzed using the two-way ANOVA of GLM procedure of SAS 9.2 (30), based on the dietary treatments and the two GIT samples (ileum and cecum). To compare microbial composition between samples and among different dietary treatments, β-diversity was measured by calculating the weighted and unweighted Unifrac distances (31) using QIIME default scripts. Principal coordinate analysis (PCoA) was applied on resulting distance matrices to generate two-dimensional plots using PRIMER v6 software [(32), PRIMER-E Ltd, Plymouth]. Permutational multivariate analysis of variance [PERMANOVA, (33)] was used to calculate *P*-values and test for significant differences of β-diversity among treatment groups.

All phyla were divided into two groups of abundance, high-abundance (≥1.0 %), and low-abundance (< 1.0 %) of the population. Differences between groups were considered significant at *P* < 0.05, and trends were considered at *P* < 0.10. Outliers were examined and removed using Grubbs' test at α < 0.05 (34). Statistical analyses for microbial sequences were performed as described by Li et al. (33) and Derakhshani et al. (20). In brief, partial least square discriminant analysis (PLS-DA; SIMCA P+ 13.0, Umetrics, Umea, Sweden) was performed on bacterial genera to identify the effects of dietary treatments. The PLS-DA is a case of partial least square regression analysis in which Y is a set of variables describing the categories of a predictor variable on X (35). In this study, X variables were bacterial taxa and Y variables were observations that belong to different dietary groups (PC, NC, or NC + FOS). For this analysis, genera which have a population lower than 0.002% were trimmed, and data were scaled using Unit Variance in SIMCA. Cross-validation was performed to determine the number of significant PLS components, and a permutation testing was conducted to validate the model. To avoid over parameterization of the model, variable influence on projection value (VIP) was estimated for each genus and genera with VIP < 0.50 were removed from the final model (35, 36).

## Results

### Sequencing Information

A total of 5,81,187 Sequences were obtained after quality filtering and chimeras checking. The minimum sequence reads were 17,411 and 14,872 for cecum and ileum samples, respectively. One ileal digesta sample from each NC and NC + FOS was identified as outlier and omitted for downstream microbial diversity and composition analyses. On average, 24,216 sequence reads per sample were generated resulting in 800 and 1,280 operational taxonomic units in the ileum and cecum digesta, respectively.

### Species Richness and Diversity

Alpha diversity indices showed difference (*P* < 0.0001) between ileal and cecal microbiota, however, no change in α-diversity was observed in response to diet or site × diet interaction (Table 1) in both ileum and cecum digesta samples.

**Table 1:** Bacterial alpha diversity based on the main effects of diet and GIT sections (ileum and cecum) of broiler chickens at 21 days of age^1^.

**Site (S)**	**Cecal digesta**			**Ileal digesta**			**SEM**	***P*** **value**		
**Diet (D)**	**PC**	**NC**	**NC + FOS**	**PC**	**NC**	**NC + FOS**		**S**	**D**	**S × D**
**ITEM**										
Shannon	6.75^a^	6.34^a^	6.42^a^	2.46^b^	3.44^b^	2.07^b^	0.458	<0.0001	0.5628	0.3472
Simpson	0.96^a^	0.94^a^	0.96^a^	0.56^b^	0.63^b^	0.52^b^	0.051	<0.0001	0.8795	0.7559
Phylogenetic Diversity	41.53^a^	37.93^a^	35.62^a^	22.60^bc^	24.77^b^	16.15^c^	2.073	<0.0001	0.0417	0.3540
Observed Species	1223.1^a^	1085.3^a^	1001.1^a^	385.9^bc^	513.3^b^	259.3^c^	82.398	<0.0001	0.0625	0.2188
Good Coverage	0.965^c^	0.969^c^	0.972^c^	0.986^ab^	0.982^b^	0.990^a^	0.002	<0.0001	0.0527	0.2319
Chao 1	2125.3^a^	1919.6^ab^	1725.6^b^	719.5^cd^	940.1^c^	563.4^d^	135.364	<0.0001	0.0487	0.2052

1 Values are the means of 4 replicate samples per treatment per site.

PC, Positive control, wheat-, corn-, and soybean meal–based diet containing adequate Ca and available P (1% Ca and 0.45% available P). NC, Negative control, wheat-, corn-, and soybean meal–based diet containing low Ca and available P (0.8% Ca and 0.25% available P). NC + FOS, NC diet supplemented with 0.5% fructooligosaccharides (FOS).

a−d Means with different superscripts within a row differ significantly (P < 0.0001)

**Figure 1:**
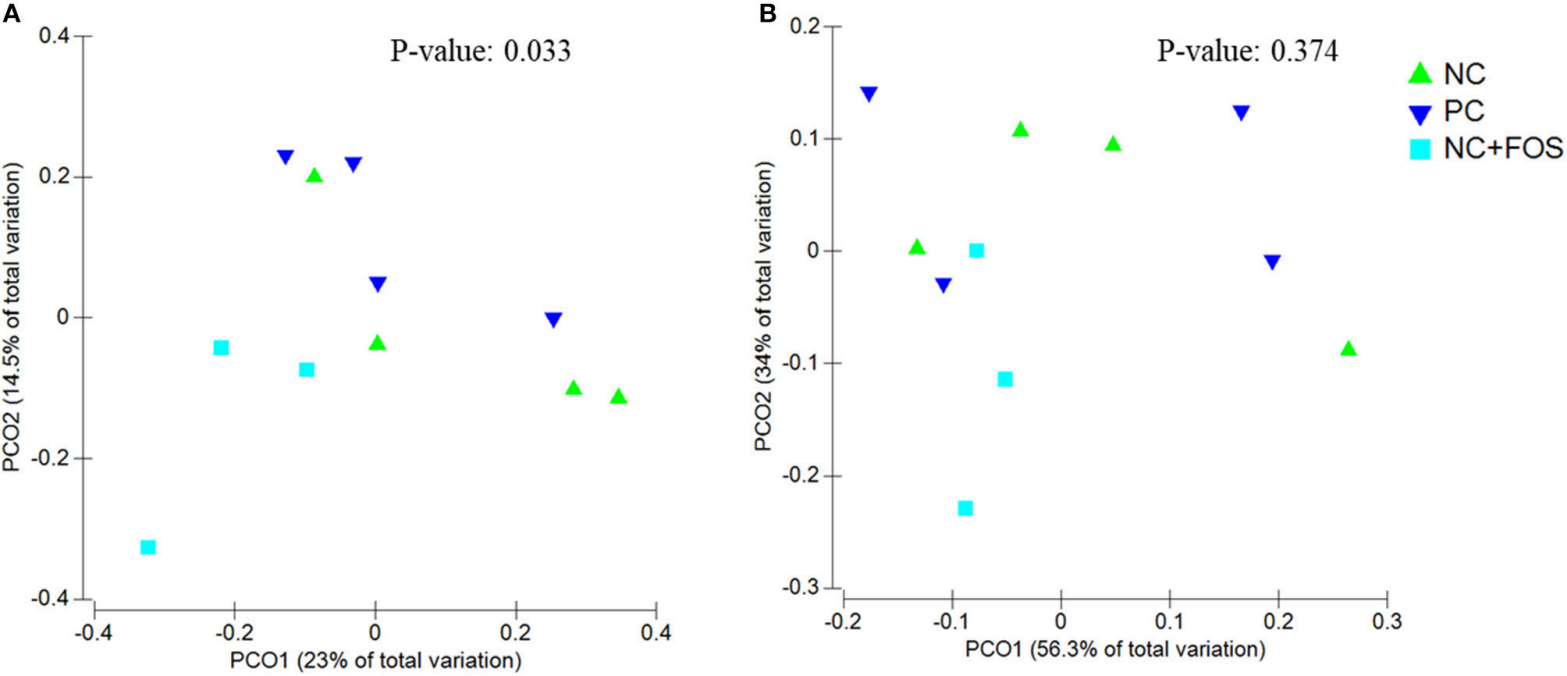
Principal coordinates analysis (PCoA) of (A) unweighted Unifrac (*P* = 0.033) and (B) weighted Unifrac (*P* = 0.374) distance of ileal digesta bacterial community between the chickens that fed PC, NC, and NC + FOS diets^1^ (n = 4/treatment). PC: Positive control, wheat-, corn-, and soybean meal–based diet containing adequate Ca and available P (1% Ca and 0.45% available P). NC: Negative control, wheat-, corn-, and soybean meal–based diet containing low Ca and available P (0.8% Ca and 0.25% available P). NC + FOS, NC diet supplemented with 0.5% fructooligosaccharides (FOS).

**Figure 2:**
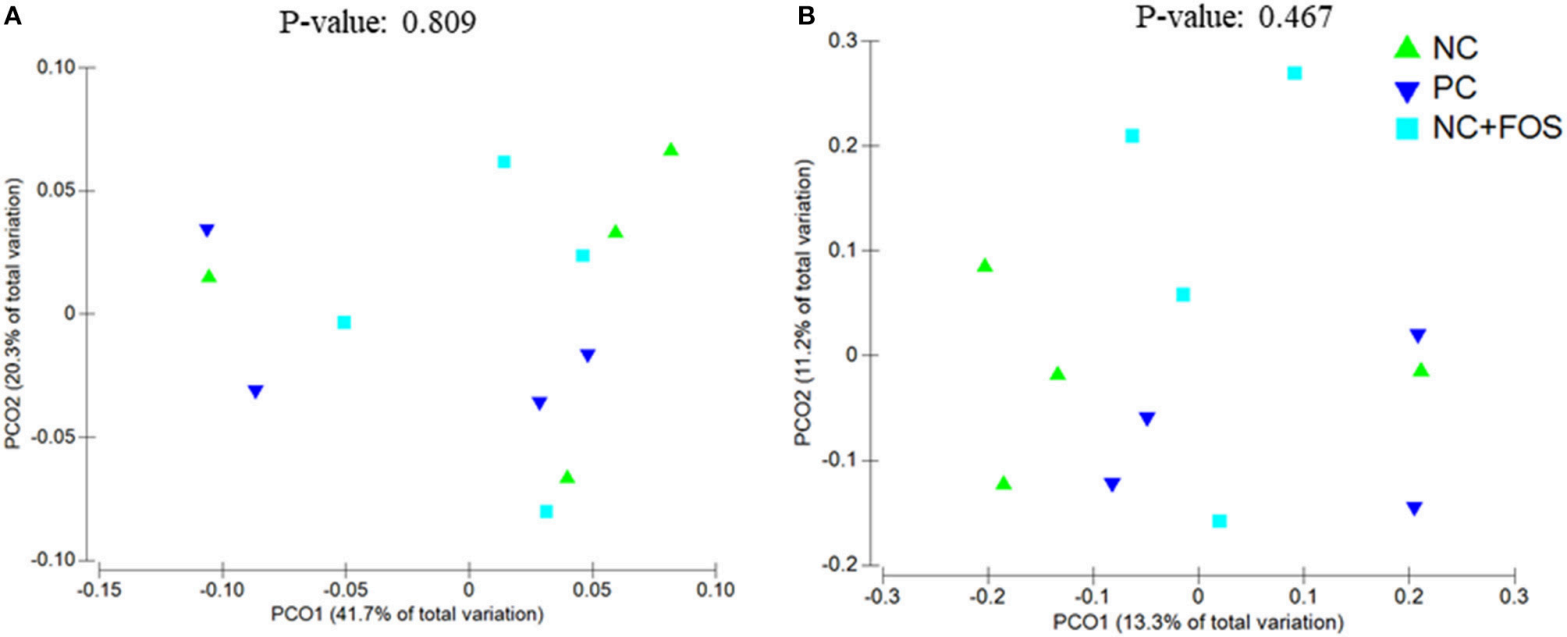
Principal coordinates analysis (PCoA) of (A) unweighted Unifrac (*P* = 0.467) and (B) weighted Unifrac (*P* = 0.809) distance of cecal digesta bacterial community between the chickens that fed PC, NC, and NC + FOS diets^1^ (*n* = 4/treatment). Refer to Figure 1 legends.

The ileal digesta of NC group showed numerically higher species richness and observed species compared to PC and NC + FOS groups (Table 1). The Principal coordinate analysis (PCoA) plot (Figure 1) analyzed using Permanova for Unifrac weighted β-diversity showed no difference (*P* > 0.05) in the ileal digesta of each treatment group. However, unweighted analysis showed difference (*P* < 0.05) between each treatment group. In the cecal digesta, the observed species and species richness (not shown) were numerically higher in PC group compared to NC and NC + FOS groups. The PCoA plot (Figure 2) analyzed using Permanova for both Unifrac weighted and unweighted β-diversity demonstrated no difference (*P* > 0.05) in cecal digesta samples in each treatment group.

### Phylogenetic Diversity

Taxonomic assignment of OTUs identified 13 phyla in the ileal digesta and 9 in the cecal digesta of the broiler chickens. Six phyla with relative abundance >0.1% are presented in Table 2. The relative abundance of most of the phyla did not differ (*P* > 0.05) in the ileum and cecum in response to diet and interaction between site and diet. However, relative abundance of phyla (Cyanobacteria, Firmicutes, and Proteobacteria) did change (*P* < 0.05) in the ileum and cecum digesta. Firmicutes had higher abundance in the cecum digesta (97%), whereas Proteobacteria (11%) and Cyanobacteria (9%) showed higher abundance in the ileum digesta. Although no difference (*P* > 0.05) in relative Firmicutes abundance was observed in the ileal digesta in different treatment groups, Firmicutes were numerically higher in abundance in the NC + FOS group followed by the NC and PC groups, whereas Proteobacteria was lower (*P* < 0.05) in the NC + FOS group.

**Table 2:** Relative abundance of bacterial phyla based on the main effects of diet and GIT sections (ileum and cecum) of broiler chickens at 21 days of age^1^.

**Site (S)**	**Cecal digesta**			**Ileal digesta**			**SEM**	***P*****-value**		
**Diet (D)**	**PC**	**NC**	**NC + FOS**	**PC**	**NC**	**NC + FOS**		**S**	**D**	**S × D**
**PHYLUM (RELATIVE ABUNDANCE %)**										
Actinobacteria	0.002^c^	0.004^c^	0.043^bc^	0.258^a^	0.131^b^	0.038^bc^	0.0233	0.0004	0.0555	0.0069
Bacteroidetes	0.049^b^	0.059^b^	0.059^b^	0.117^a^	0.097^ab^	0.092^ab^	0.0082	0.0046	0.9150	0.5717
Cyanobacteria	0.000^b^	0.000^b^	0.000^b^	10.196^a^	9.916^a^	6.692^ab^	1.3042	<0.0001	0.7600	0.7600
Firmicutes	97.175^a^	97.379^a^	95.560^a^	73.346^b^	80.427^b^	86.308^ab^	2.5055	0.0001	0.4231	0.2670
Proteobacteria	0.260^c^	0.413^c^	2.237^bc^	15.909^a^	9.209^ab^	6.461^bc^	1.4568	<0.0001	0.2196	0.0658
Tenericutes	1.990^a^	1.189^ab^	1.360^ab^	0.043^b^	0.0516^b^	0.006^b^	0.2455	0.0023	0.6980	0.7038
Unclassified	0.520^ab^	0.948^a^	0.735^ab^	0.120^b^	0.157^b^	0.129^b^	0.0990	0.0018	0.4979	0.6072

1 Values are the means of 4 replicate samples per treatment per site.

PC, Positive control, wheat-, corn-, and soybean meal–based diet containing adequate Ca and available P (1% Ca and 0.45% available P). NC, Negative control, wheat-, corn-, and soybean meal–based diet containing low Ca and available P (0.8% Ca and 0.25% available P). NC + FOS, NC diet supplemented with 0.5% fructooligosaccharides (FOS).

a−d *Means with different superscripts within a row differ significantly (P < 0.0001)*.

### Dietary Effect on Ileal Microbiota

**Figure 3:**
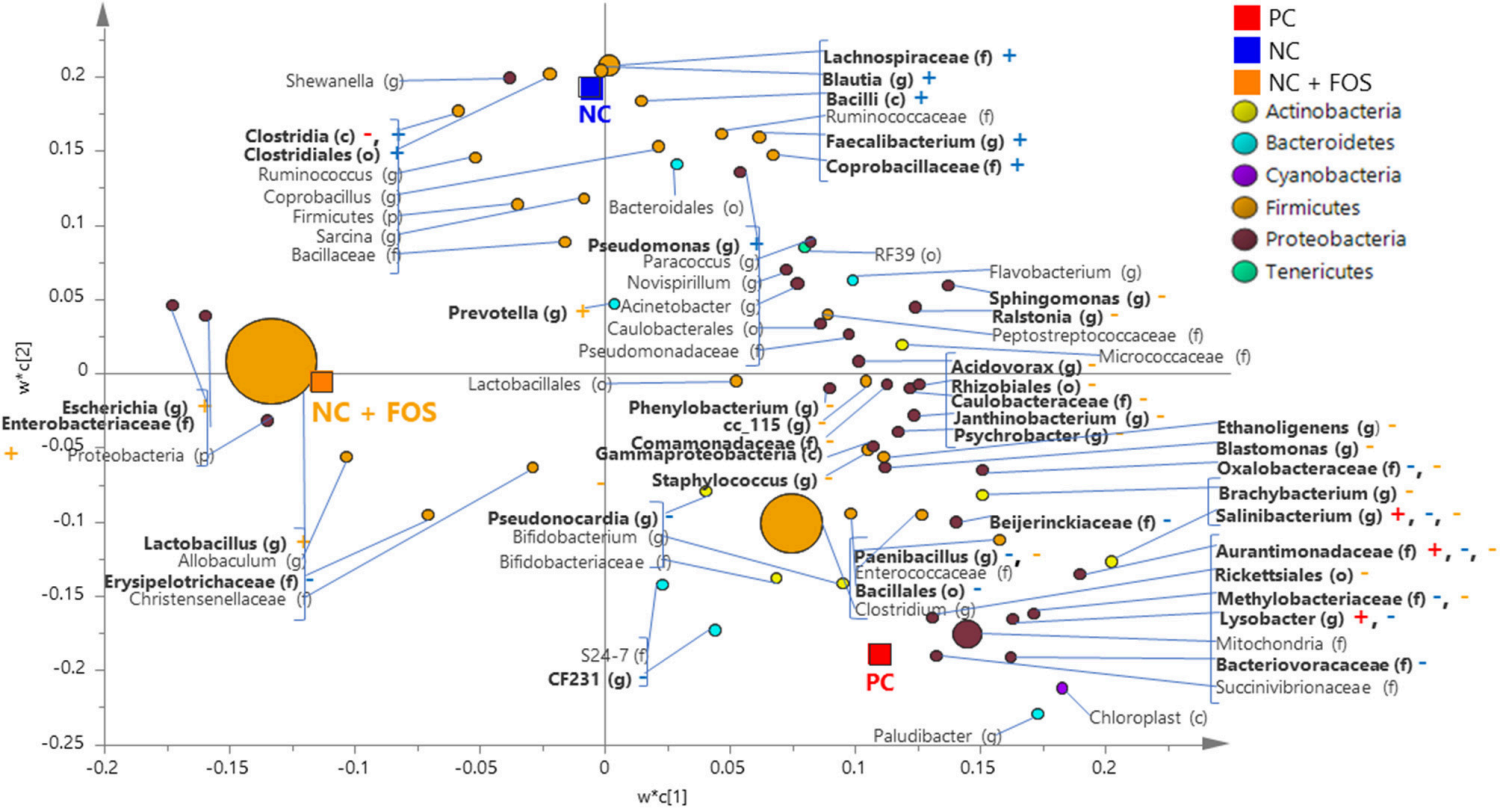
Partial least square discriminant analysis (PLS-DA) loading plot based on the relative abundance of bacterial taxa in the ileal digesta of broiler chickens that fed PC, NC, and NC + FOS diets^1^ (*n* = 4/treatment). The presenting taxa are chosen at variable influence on projection (VIP) value of above 0.6. The size of each circle indicates the abundance of taxa and is colored according to their corresponding phyla. The taxa are presented on phylum (p), class (c), order (o), family (f), or genus (g) levels after comparison of sequences to the Greengenes Core reference database. The colored “+” and “–” indicates positive or negative correlation of taxa to the same colored PC, NC, or NC + FOS dietary group. Refer to Figure 1 legends.

In the ileal digesta of broiler chickens, there were 75 bacterial taxa shared across the three dietary treatments (PC, NC, and NC + FOS). Eight genera were unique to NC + FOS group, whereas 28 and 18 genera were unique to PC and NC groups, respectively (Supplementary Figure 1). The taxa shared between all the three groups include *Salinibacterium* and Aurantimonadaceae (f). Clostridia were found in the PC and NC groups but not observed in the NC +FOS group. As shown on the PLS-DA loading plot (Figure 3), the genus *Salinibacterium* and *Lysobacter* were positively correlated to the ileal digesta of broiler chickens fed PC diet. Comparison between the NC and NC + FOS groups in the ileal digesta by PLS-DA (Figure 3) indicated that Clostridia, *Blautia, Faecalibacterium*, and *Pseudomonas* were associated with the NC group, whereas *Escherichia, Lactobacillus, Prevotella* were positively correlated to the NC + FOS group.

### Dietary Effect on Cecal Digesta Microbiota

**Figure 4:**
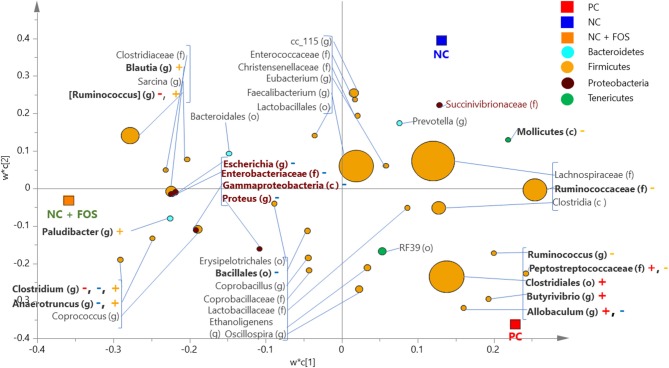
Partial least square discriminant analysis (PLS-DA) loading plot based on the relative abundance of bacterial taxa in the cecal digesta of broiler chickens that fed PC and NC + FOS diets^1^ (*n* = 4/treatment). The presenting taxa are chosen at variable influence on projection (VIP) value of above 0.5. The size of each circle indicates the abundance of taxa and is colored according to their corresponding phyla. The taxa are presented on phylum (p), class (c), order (o), family (f), or genus (g) levels after comparison of sequences to the Greengenes Core reference database. The colored “+” and “–” indicates positive or negative correlation of taxa to the same colored PC or NC + FOS dietary group. Refer to Figure 1 legends.

In the cecal digesta of broiler chickens, 65 bacterial taxa were shared among the three dietary treatments as shown in Supplementary Figure 2. Seven genera, such as *Paludibacter*, Clostridium, *Blautia, Coprococcus, Coprobacillus, Ethanoligenes*, and *Oscillospira*, were unique to the NC + FOS group. The PLS-DA comparison (at cut-off VIP value of 0.5) between PC and NC + FOS groups in the cecal digesta of broiler chickens (Figure 4) showed that members of Peptostreptococcaceae (f), Clostridiales (o) and genus *Butyrivibrio* and *Allobaculum* were positively associated with PC, whereas *Blautia* and *Ruminococcus* (g) exhibited positive association with NC + FOS.

## Discussion

Low Ca and aP level with and without FOS supplementation could potentially affect the structure of the microbial community in the digestive tract. In this study, cecal and ileal digesta were analyzed to evaluate the difference of microbial community in response to low Ca, aP, and FOS supplemented diets. The ileum and cecum sections contain diverse microbial populations that are responsible for degrading complex organic molecules to short chain fatty acids (SCFAs), such as the feed-based phytate degrading activities (37–40). On the other hand, the cecum has greater species richness and diversity compared to the ileum, and this complexity increases as the chicks advances in age (41–46).

Our findings were in accordance with previous reports that the cecum had greater species richness compared to the ileum (47, 48). However, no difference in diversity was observed with a low Ca and aP and/ or FOS dietary supplementation. Any dietary changes remarkably affect the richness of microbial community in the ceca compared to ilea (with shorter transit time for digesta, 3.5 h). Increased abundance of Clostridia in chicken ileal (PC and NC diets) and cecal digesta indicates a healthy chicken gut as members of Clostridial group plays an important role in SCFA metabolism (40). In addition to immunomodulatory and nutritional functions, SCFA are known for mineral absorption and inhibition of pathogenic microbes by reducing pH (49). Furthermore, genus *Clostridium* is associated with the production of cysteine phytase, one of the four distinct classes of phytate-degrading phytases. Although phytases are structurally different but they all have similar functions (50). Abundance of *Clostridium* in the ileum and cecum may increase the endogenous bacterial phytase production and thus potentially help improving the P availability, mineral absorption, ileal amino-acid digestibility, and thus can improve bird performance. However, their role depends on the levels of P and Ca in the diet.

Difference in α-diversity indices and phylogenetic community between the ileal and cecal digesta confirmed the results of Borda-Molina and coworkers (51) who reported greater distinction (*p* = 0.001) in bacterial community between the ileum and cecum samples, regardless of the diet. Similar results were also obtained by Stanley et al. (52) and Witzig et al. (53). The difference in microbial diversity of the cecal and ileal digesta can be attributed to many factors such as physiochemical conditions, passage rate of the digesta, pH, presence and absence of small and soluble particles. These conditions help in the establishment of complex microbiota and enhance their role in nutrient assimilation, vitamins, and amino acids production (54, 55), and pathogen elimination (52). Most of the colonized microorganisms in the two GIT sections belonged to phylum Firmicutes, which have been commonly described in earlier reports of the chicken GIT (38, 41, 51, 56, 57). The microbial community differences observed in the ileum of all three dietary groups (PC, NC, and NC + FOS) were in agreement with earlier study (51). It has been reported that low doses of Ca in the diets (NC and NC + FOS) can lead to a decrease in pH (2) and enhance the pre-cecal digestibility (58, 59) which lead to positive association of lactobacilli in the ileum (2, 51). Moreover, increased availability of P could increase the abundance of beneficial microbes such as *Blautia* and *Faecalibacterium*, which agrees with the results of Borda-Molina et al. (51). Genus *Faecalibacterium*, is known for its function of epithelial health enhancement, by increasing duodenal villus/crypt ratio (60), and for butyrate production (61). In addition, *Faecalibacterium* regulates inflammatory gene expressions and apoptosis in host cells (62). *Blautia*, a member of family Lachnospiraceae (63) and it constitutes one of the major taxonomic groups of the human/rumen/chicken gut microbiota where they are associated with corn-based diet and can degrade complex polysaccharides for energy utilization by the host (64, 65).

Supplementation of FOS along with the NC diet exhibited positive association with the genus *Lactobacillus* and *Prevotella* in the ileum. Earlier studies in chickens, mice and pigs have also shown that diets supplemented with P increased the *Lactobacillus* abundance (51, 57, 66–68). Further supplementation of dietary FOS has been known to selectively supporting the growth of *Lactobacillus*. Lactobacilli produces extracellular enzymes to degrade FOS (69) which results in an increase in SCFAs and lactate production, which could further enhance the immune system (70–72). Additionally, it has also been reported that a high availability of FOS could also be associated with the oligosaccharide transport system of the *Lactobacillus* species (69, 73, 74). Some gut microbes such as Prevotella also have prebiotic degrading capability, as shown in our study (69). Genus *Prevotella* are known for their oligosaccharides degrading property. Additionally, several species of *Prevotella* possess dipeptidyl peptidase type IV which is important for initial dietary protein catabolic activity (75, 76). These mechanisms positively correlate the abundance of *Prevotella* in wheat-corn based diets, as evident in this study. In a recent study, Poeker et al. (77) also demonstrated that fermentable dietary fibers can induce growth and/ or activity of specific beneficial populations. The abundance of Prevotella and Lactobacillus in NC + FOS fed broilers again indicates healthy gut with higher SCFA production and improved mineral absorption. Although PC fed groups showed presence of healthy bacteria, but to understand the abundance of Lysobacter and Salinibacterium and their attributed function in PC fed group. These identified functional attributes are descriptive in nature, therefore it is unclear if the modulated bacteria confer benefits to the host, if they are artifacts or if they are markers of a modulated gut bacteria that helps in well-being of the host. This study was limited to chicken cecum and ileum, future work should also include locations in the upper GI tract to determine the microbial changes that occur there, since they are also likely important in the overall health and performance of the poultry. Nevertheless, this study provides a more comprehensive glimpse at the gut microbial modulations in response to low Ca and P in broilers diet and provides future targets and markers while using alternative approaches with FOS and Ca and P levels in diets.

In conclusion, a low dietary Ca and aP level with or without FOS supplementation could influence the microbial community of the chicken gut. Diets supplemented with a low Ca and aP, affected the ileum microbiota, whereas the FOS supplementation led to an increased abundance of beneficial microbes such as *Lactobacillus, Faecalibacterium, Blautia, Prevotella* etc. Increased abundance of *Clostridium* in the cecum may be an evidence that common core members of the gut microbiota also have the ability to produce phytase which can further improve the availability of P in the chicken diet. However, to better understand the effect of low Ca, aP diet and FOS supplementation on the growth performance and gut health of broiler chickens, further mucosal microbial community studies are warranted. Moreover, in order to overcome the restrictions of 16S rRNA based microbial community analysis, metaproteomics, and transcriptomics are needed to determine the metabolic and functional properties of the chicken gut microbiota.

## Ethics Statement

The study was conducted out in strict accordance with the guidelines established by the Canadian Council on Animal Care (78). All the experimental procedures were approved by the University of Manitoba Animal Care Protocol Management and Review Committee.

## Author Contributions

WK brought research idea, designed the study, managed the project, and contributed to data interpretation and manuscript writing. SK analyzed the data, interpreted the data, and wrote the manuscript. YS conducted the study, collected the data, analyzed the data, and contributed to data interpretation and manuscript writing.

### Conflict of Interest Statement

The authors declare that the research was conducted in the absence of any commercial or financial relationships that could be construed as a potential conflict of interest.

## Supplementary Material

Supplementary Figure 1 Venn diagram of shared and unique bacterial taxa in ileal digesta of broiler chickens fed PC, NC, and NC + FOS diet^1^ (*n* = 4/treatment). See Figure 1 legends.

Supplementary Figure 2 Venn diagram of shared and unique bacterial taxa in cecal digesta of broiler chickens fed PC, NC, and NC + FOS diet^1^ (*n* = 4/treatment). See Figure 1 legends.

Supplementary Table 1 Composition of experimental diets (as-fed basis).
