# Supplementary figures and images for: Insight Into Dynamics of Gut Microbial Community of Broilers Fed With Fructooligosaccharides Supplemented Low Calcium and Phosphorus Diets
Source: Front Vet Sci. 2019 Mar 29;6:95. doi: 10.3389/fvets.2019.00095 (PMC6449842; doi:10.3389/fvets.2019.00095)

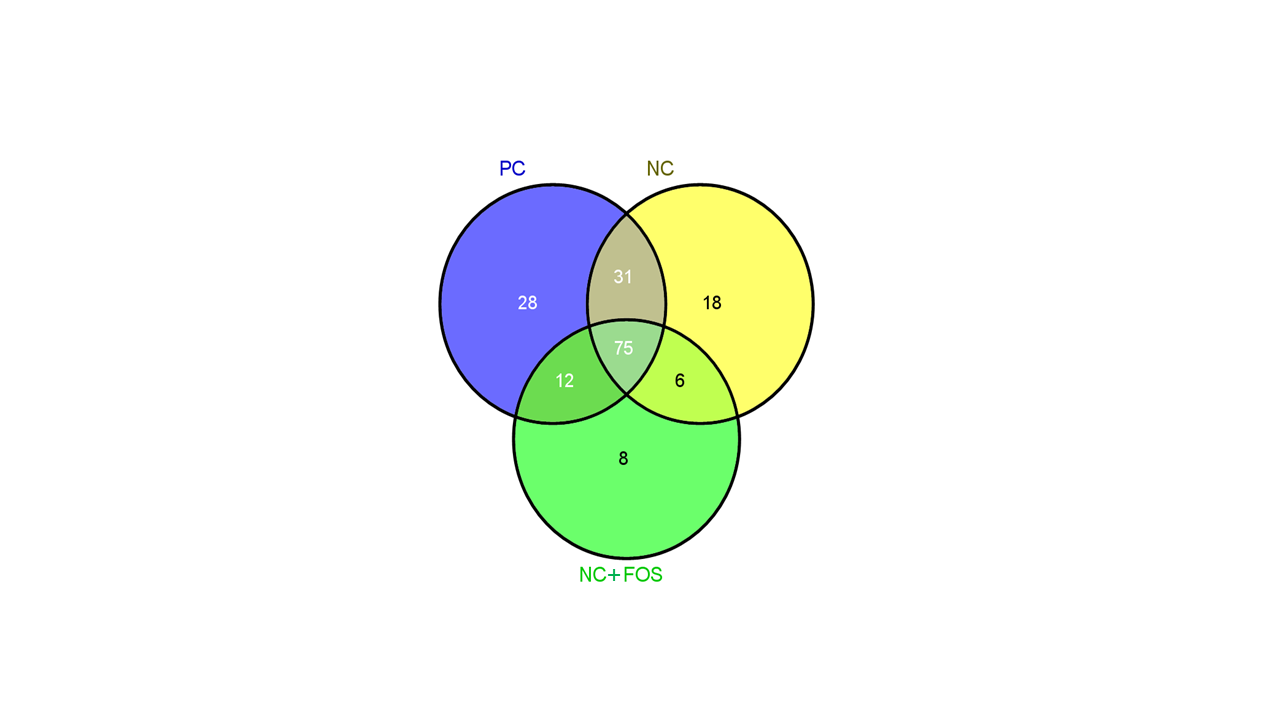

Supplement: Supplementary Figure 1 — Venn diagram of shared and unique bacterial taxa in ileal digesta of broiler chickens fed PC, NC, and NC + FOS diet1 (n = 4/treatment). See Figure 1 legends. [file Image_1.TIF]

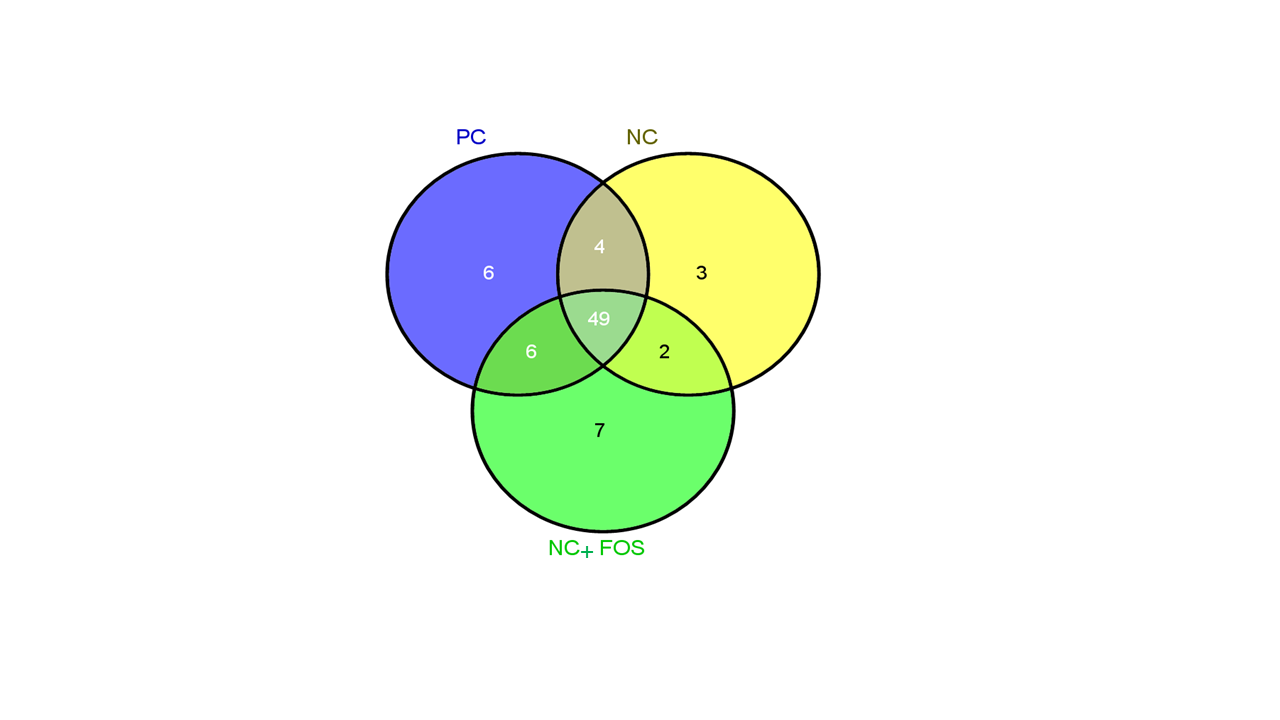

Supplement: Supplementary Figure 2 — Venn diagram of shared and unique bacterial taxa in cecal digesta of broiler chickens fed PC, NC, and NC + FOS diet1 (n = 4/treatment). See Figure 1 legends. [file Image_2.TIF]
