# Supplementary material for: Insight Into Dynamics of Gut Microbial Community of Broilers Fed With Fructooligosaccharides Supplemented Low Calcium and Phosphorus Diets
Source: Front Vet Sci. 2019 Mar 29;6:95. doi: 10.3389/fvets.2019.00095 (PMC6449842; doi:10.3389/fvets.2019.00095)
Supplement: Supplementary Table 1 — Composition of experimental diets (as-fed basis). [file Table_1.pdf]

Supplementary Table 1. Composition of experimental diets (as-fed basis)

Item	Diet		
	**PC[1]**	**NC[2]**	**NC + 0.5% FOS**
**Ingredient (% of diet)**			
FOS[3]	-	-	0.5
Wheat	35.80	36.00	35.26
Corn	29.80	31.45	31.45
Soybean meal	20.46	19.36	19.36
Canola meal	4.25	5.00	4.72
Canola Oil	4.50	4.00	4.00
Limestone	1.38	1.46	1.46
Dicalcium phosphate	1.76	0.65	0.65
DL-Methionine	0.10	0.10	0.10
L-Lysine HCl	0.12	0.14	0.14
Threonine	0.04	0.04	0.04
Mineral premix[4]	0.50	0.50	0.50
Vitamin premix[5]	1.00	1.00	1.00
Titanium dioxide[6]	0.30	0.30	0.30
**Calculated composition[7]**			
ME (kcal/kg)	3,111	3,119	3,105
CP (%)	21.3	21.3	21.2
Ca (%)	1.00	0.80	0.80
Available P (%)	0.45	0.25	0.25
Met + Cys (%)	0.97	0.98	0.98
Met (%)	0.50	0.50	0.50
Lys (%)	1.08	1.08	1.08
Thr (%)	0.80	0.80	0.80
**Analyzed composition**			
CP (%)	21.0	20.7	20.6
Ca (%)	1.24	0.98	0.95
Total P (%)	0.73	0.54	0.56
Available P (%)	0.45	0.23	0.26
DM (%)	90.3	89.9	89.9

[1]PC: Positive control, wheat-, corn-, and soybean meal–based diet containing adequate Ca and available P (1% Ca and 0.45% available P).

[2]NC: Negative control, wheat-, corn-, and soybean meal–based diet containing low Ca and available P (0.8% Ca and 0.25% available P).

[3]Nutraflora P-95, Short-Chain Fructooligosaccharides (scFOS), contains 4.5% sugar (fructose + glucose + sucrose), 34.2% GF2 (glucose + 2 molecules fructose), 48.9% GF3 (glucose + 3 molecules fructose), and 12.4% GF4 (glucose + 4 molecules fructose) on DM basis (Ingredion, Etobicoke, ON, Canada).

[4]Supplied per kilogram of diet: Mn, 70 mg; Zn, 80 mg; Fe, 80 mg; Cu, 10 mg; Se, 0.3 mg; I, 0.5 mg; and NaCl, 4.3 g.

[5]Supplied per kilogram of diet: vitamin A, 8,250 IU; vitamin D3, 3,000 IU; vitamin E, 30 IU; vitamin B12, 0.013 mg; vitamin K, 2 mg; riboflavin, 6 mg; pantothenic acid, 11 mg; niacin, 41.6 mg; choline, 1,300.8 mg; folic acid, 4 mg; biotin, 0.25 mg; pyridoxine, 4 mg; thiamine, 4 mg; endox (antiox), 125 mg; DL-methionine, 500 mg; virginiamycin (Stafac-22), 11mg; and monensin sodium (Coban), 99 mg.

[6]Aldrich- 248576 (Sigma-Aldrich Co. LLC, ON, Canada).

[7]Concentrations were calculated based on NRC (1994) guidelines
